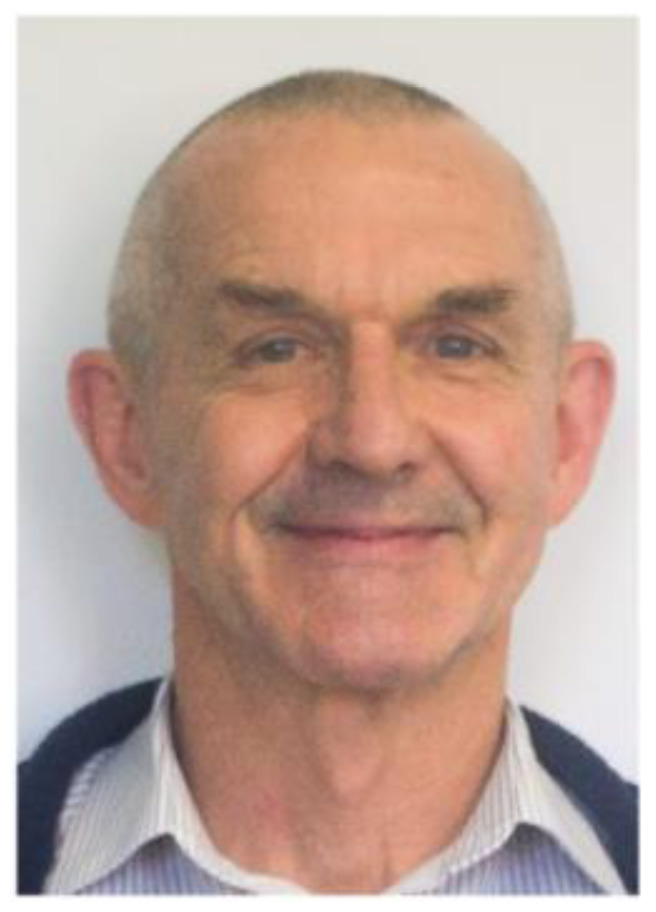# South African Journal of Sports Medicine: What happened in 2020 and plans for 2021

**DOI:** 10.17159/2078-516X/2021/v33i1a9378

**Published:** 2021-01-15

**Authors:** Mike Lambert

**Affiliations:** Editor-in-chief

It has become cliché to comment on the hardships most people faced as a consequence of the pandemic in 2020. Without trivialising these hardships, the South African Journal of Sports Medicine had a bumper year. The published outputs of the journal doubled in 2020 compared to the number published in each of the previous two years. There were 7105 downloads of papers published in 2020. The most popular paper based on number of downloads was “*Implications of COVID-19 for resumption of sport in South Africa: A South African Sports Medicine Association (SASMA) position statement - Part 1*” with 653 downloads; the abstract for this paper was viewed 1537 times.^[1]^ The paper “*Physical and physiological profile of U18, U19, U21 and senior elite netball players*”^[2]^ had 514 downloads and the paper “*Brace yourselves: esports is coming”* was the third most popular paper with 424 downloads.^[3]^ The broad interest in physical activity and sport and Covid-19 was not neglected. Two commentaries on Covid-19, two position statements compiled by the South African Sports Medicine Association and one short report on the wearing of face masks during exercise were published. These outputs on Covid-19 have contributed to the public message and guidelines for exercising and playing sport during the pandemic.

In addition to the traditional metrics quantifying the uptake of papers, altmetric data can also be used to assess the journal’s impact. Altmetrics assess data from the internet such as mentions made on social networks, Wikipedia and public policy documents. These data provide another dimension for assessing how a paper has been received. According to these criteria, the paper “*Testosterone boosters: a report of a supplement’s misleading labelling claims*” ^[4]^ has the highest score followed by the “*The Currie Cup Premiership Competition Injury Surveillance Report 2019*”. ^[5]^ There were 537 tweets on the published content of the South African Journal of Sports Medicine in 2020. These tweets were posted by 234 unique tweeters from 26 countries. Besides South Africa, most of the tweeters were from the United Kingdom, Ireland, Australia and the United States. There were 12 news stories about the content of the journal in 2020 communicated by seven unique news outlets in five countries (Australia, United States, Zimbabwe, Turkey and New Zealand). Another statistic is that authors from the University of the Witswatersrand had the most outputs, followed by the University of Cape Town, and University of Pretoria.

Much of the success of the journal can be attributed to the high quality reviews of the submitted papers. The pool of dedicated reviewers invited to review papers submitted to the South African Journal of Sports Medicine invested much time in trying to improve the quality of the paper they were asked to review. In some cases the reviewers interacted with the revised paper up to four times before it got accepted for publication. Although this is frustrating for the authors, there is no doubt that the final published version is vastly improved compared to their original submitted paper. Reviewing is a thankless task for which there is no monetary reward or public recognition. Most journals find it difficult to find reviewers. However, this has not been a major obstacle for the South African Journal of Sports Medicine.

The South African Journal of Sports Medicine has also been fortunate to be supported by the Academy of Science of South Africa (ASSAf) who have provided the infrastructure for the journal’s website. They are quick to respond to any queries related to the Open Journal Systems (OJS), the software application used for the online presence of the journal. ASSAf have also hosted workshops for editors and journal managers to ensure they remain up-to-date with the new developments in the academic publishing world. The South African Journal of Sports Medicine remains accredited by the Department of Higher Education and Training. Therefore authors from South African academic institutions can claim their subsidy after their paper is published. However, the subsidies are dealt with differently at each institution.

While it is important to look back and acknowledge accomplishments, this should be balanced with looking forward to plan goals. With that said the journal has an exciting year ahead. The first goal is to increase the number of outputs and downloads in 2021. Several papers are already in an advanced stage of review, so the year should get off to a good start. The second goal is to get the journal listed by the Web of Science database. This is a challenging task in which the current and past quality of the papers published by the South African Journal of Sports Medicine are reviewed by a panel. Achieving both goals will raise the journal to the next level of accomplishment.

The South African Journal of Sports Medicine will also be working with the organisers of the International Festival of Sports and Exercise Medicine Conference to publish their peer-reviewed conference abstracts. This conference represents the South African Society of Biomechanics, South African Sports Medicine Association, Life Through Movement international Conference and The African Physical Activity Network. The conference will be held at the CSIR International Convention Centre in Pretoria from the 22–24th October 2021.

Let the games begin![Fig f1-2078-516x-33-v33i1a9378]

**Figure f1-2078-516x-33-v33i1a9378:**